# Global data set of long-term summertime vertical temperature profiles in 153 lakes

**DOI:** 10.1038/s41597-021-00983-y

**Published:** 2021-08-04

**Authors:** Rachel M. Pilla, Elizabeth M. Mette, Craig E. Williamson, Boris V. Adamovich, Rita Adrian, Orlane Anneville, Esteban Balseiro, Syuhei Ban, Sudeep Chandra, William Colom-Montero, Shawn P. Devlin, Margaret A. Dix, Martin T. Dokulil, Natalie A. Feldsine, Heidrun Feuchtmayr, Natalie K. Fogarty, Evelyn E. Gaiser, Scott F. Girdner, María J. González, K. David Hambright, David P. Hamilton, Karl Havens, Dag O. Hessen, Harald Hetzenauer, Scott N. Higgins, Timo H. Huttula, Hannu Huuskonen, Peter D. F. Isles, Klaus D. Joehnk, Wendel Bill Keller, Jen Klug, Lesley B. Knoll, Johanna Korhonen, Nikolai M. Korovchinsky, Oliver Köster, Benjamin M. Kraemer, Peter R. Leavitt, Barbara Leoni, Fabio Lepori, Ekaterina V. Lepskaya, Noah R. Lottig, Martin S. Luger, Stephen C. Maberly, Sally MacIntyre, Chris McBride, Peter McIntyre, Stephanie J. Melles, Beatriz Modenutti, Dörthe C. Müller-Navarra, Laura Pacholski, Andrew M. Paterson, Don C. Pierson, Helen V. Pislegina, Pierre-Denis Plisnier, David C. Richardson, Alon Rimmer, Michela Rogora, Denis Y. Rogozin, James A. Rusak, Olga O. Rusanovskaya, Steve Sadro, Nico Salmaso, Jasmine E. Saros, Jouko Sarvala, Émilie Saulnier-Talbot, Daniel E. Schindler, Svetlana V. Shimaraeva, Eugene A. Silow, Lewis M. Sitoki, Ruben Sommaruga, Dietmar Straile, Kristin E. Strock, Hilary Swain, Jason M. Tallant, Wim Thiery, Maxim A. Timofeyev, Alexander P. Tolomeev, Koji Tominaga, Michael J. Vanni, Piet Verburg, Rolf D. Vinebrooke, Josef Wanzenböck, Kathleen Weathers, Gesa A. Weyhenmeyer, Egor S. Zadereev, Tatyana V. Zhukova

**Affiliations:** 1 Miami University, Department of Biology, Oxford, Ohio USA; 2 Belarusian State University, Faculty of Biology, Minsk, Belarus; 3 Leibniz-Institute of Freshwater Ecology and Inland Fisheries, Department of Ecosystem Research, Berlin, Germany; 4 INRAE, University of Savoie Mont-Blanc, CARRTEL, Thonon-les-Bains, France; 5 University of Comahue: INIBIOMA, CONICET, Neuquén, Argentina; 6 University of Shiga Prefecture, Hikone, Shiga, Japan; 7 University of Nevada, Reno, Global Water Center, Reno, Nevada USA; 8 Uppsala University, Department of Ecology and Genetics/Limnology, Uppsala, Sweden; 9 University of Montana, Flathead Lake Biological Station, Polson, Montana, USA; 10 Universidad del Valle de Guatemala Centro de Estudios Atitlan, Guatemala, Guatemala; 11 University of Innsbruck, Research Department for Limnology Mondsee, Mondsee, Austria; 12 Mohonk Preserve, Daniel Smiley Research Center, New Paltz, New York USA; 13 UK Centre for Ecology & Hydrology, Lake Ecosystems Group, Lancaster, UK; 14 Seqwater, Ipswich, QLD Australia; 15 Florida International University, Department of Biological Sciences and Institute of Environment, Miami, Florida USA; 16 U.S. National Park Service, Crater Lake National Park, Crater Lake, Oregon USA; 17 University of Oklahoma, Department of Biology, Norman, Oklahoma USA; 18 Griffith University, Australian Rivers Institute, Nathan, Australia; 19 University of Florida, Gainesville, Florida USA; 20 University of Oslo, Department of Biosciences, Oslo, Norway; 21 LUBW Landesanstalt für Umwelt, Messungen und Naturschutz Baden-Württemberg, Institut für Seenforschung, Langenargen, Germany; 22 IISD Experimental Lake Area Inc., Winnipeg, Manitoba Canada; 23 FAO, BELSPO, Brussels, Belgium; 24 University of Eastern Finland, Department of Environmental and Biological Sciences, Joensuu, Finland; 25 Swiss Federal Institute of Aquatic Science and Technology, Department of Aquatic Ecology, Dübendorf, Switzerland; 26 CSIRO, Land and Water, Canberra, Australia; 27 Laurentian University, Cooperative Freshwater Ecology Unit, Sudbury, Ontario, Canada; 28 Fairfield University, Biology Department, Fairfield, Connecticut USA; 29 University of Minnesota, Itasca Biological Station and Laboratories, Lake Itasca, Minnesota USA; 30 Finnish Environment Institute SYKE, Freshwater Center, Helsinki, Finland; 31 A.N. Severtsov Institute of Ecology and Evolution of The Russian Academy of Sciences, Laboratory of Ecology of Water Communities and Invasions, Moscow, Russia; 32 Zurich Water Supply, City of Zurich, Zurich, Switzerland; 33 University of Regina, Institute of Environmental Change and Society, Regina, Saskatchewan Canada; 34 Milano-Bicocca University, Milan, Italy; 35 University of Applied Sciences and Arts of Southern Switzerland, Department for Environment, Constructions and Design, Canobbio, Switzerland; 36 Kamchatka Research Institute of Fisheries & Oceanography, now Kamchatka Branch of Russian Federal Research Institute of Fisheries and Oceanography, Petropavlovsk-Kamchatsky, Russia; 37 University of Wisconsin, Center for Limnology, Boulder Junction, Wisconsin USA; 38 Federal Agency for Water Management, Institute for Aquatic Ecology and Fisheries Management, Mondsee, Austria; 39 University of California Santa Barbara, Department of Ecology, Evolution and Marine Biology, Santa Barbara, California, USA; 40 University of Waikato, Environmental Research Institute, Hamilton, New Zealand; 41 Ryerson University, Department of Chemistry and Biology, Toronto, Ontario Canada; 42 University of Hamburg, Department of Biology, Hamburg, Germany; 43 Dominion Diamond Mines, Environment Department, Calgary, Alberta Canada; 44 Ontario Ministry of the Environment, Conservation and Parks, Dorset Environmental Science Centre, Dorset, Ontario Canada; 45 Irkutsk State University, Institute of Biology, Irkutsk, Russia; 46 University of Liège, Chemical Oceanography Unit, Institut de Physique (B5A), Liège, Belgium; 47 SUNY New Paltz, Biology Department, New Paltz, New York USA; 48 The Kinneret Limnological Laboratory, Israel Oceanographic and Limnological Research, Migdal, Israel; 49 CNR Water Research institute, Verbania, Verbania, Pallanza Italy; 50 Krasnoyarsk Scientific Center SB RAS, Institute of Biophysics, Krasnoyarsk, Russia; 51 University of California Davis, Department of Environmental Science and Policy, Davis, California USA; 52 Fondazione Edmund Mach, Research and Innovation Centre, San Michele all’Adige, Italy; 53 University of Maine, Climate Change Institute, Orono, Maine USA; 54 University of Turku, Turku, Finland; 55 Université Laval, Departments of Biology and Geography, Québec, Canada; 56 University of Washington, School of Aquatic and Fishery Sciences, Seattle, Washington USA; 57 The Technical University of Kenya, Department of Geosciences and the Environment, Nairobi, Kenya; 58 University of Innsbruck, Department of Ecology, Innsbruck, Austria; 59 University of Konstanz, Limnological Institute, Konstanz, Germany; 60 Dickinson College, Department of Environmental Science, Carlisle, Pennsylvania USA; 61 Archbold Biological Station, Venus, Florida USA; 62 University of Michigan, Biological Station, Pellston, Michigan USA; 63 Vrije Universiteit Brussel, Department of Hydrology and Hydraulic Engineering, Brussels, Belgium; 64 ETH Zurich, Institute for Atmospheric and Climate Science, Zurich, Switzerland; 65 National Institute of Water & Atmospheric Research, Hamilton, New Zealand; 66 University of Alberta, Department of Biological Sciences, Edmonton, Alberta Canada; 67 Cary Institute of Ecosystem Studies, Millbrook, New York USA

**Keywords:** Freshwater ecology, Limnology, Freshwater ecology

## Abstract

Climate change and other anthropogenic stressors have led to long-term changes in the thermal structure, including surface temperatures, deepwater temperatures, and vertical thermal gradients, in many lakes around the world. Though many studies highlight warming of surface water temperatures in lakes worldwide, less is known about long-term trends in full vertical thermal structure and deepwater temperatures, which have been changing less consistently in both direction and magnitude. Here, we present a globally-expansive data set of summertime *in-situ* vertical temperature profiles from 153 lakes, with one time series beginning as early as 1894. We also compiled lake geographic, morphometric, and water quality variables that can influence vertical thermal structure through a variety of potential mechanisms in these lakes. These long-term time series of vertical temperature profiles and corresponding lake characteristics serve as valuable data to help understand changes and drivers of lake thermal structure in a time of rapid global and ecological change.

## Abstract (summary)

**Table:** 

Measurement(s)	temperature of water • temperature profile
Technology Type(s)	digital curation
Factor Type(s)	lake location • temporal interval
Sample Characteristic - Environment	lake • reservoir
Sample Characteristic - Location	global

Machine-accessible metadata file describing the reported data: 10.6084/m9.figshare.14619009

## Background & Summary

Lakes serve as important sentinels of climate and environmental changes^1,2^, and also as sources of vital ecosystem services, such as fresh drinking water and fisheries. Several recent regional- to global-scale studies have quantified generally consistent trends of warming surface waters^3,4^, though few studies at broad geographic scales have considered changes in the full vertical thermal structure of lakes^5–7^. Changes in vertical thermal structure can affect ecological processes in lakes at depth, including vertical mixing^8,9^, oxygen depletion^10,11^, and productivity^12^. Further, deep waters are areas of critical habitat for many species, and changes in vertical thermal structure at depth can alter population dynamics or trophic interactions based on the quality and availability of suitable habitat^13–15^.

Drivers of vertical lake thermal structure may include those most important to surface water temperature, including air temperature^3,4^, shortwave and longwave radiation^16^, wind speed^17^, and relative humidity^18^. However, a more complex interaction exists between atmospheric meteorological drivers and vertical thermal structure, which underscores the importance of other factors to understanding trends in deepwater temperatures and vertical thermal structure. For example, water clarity is particularly influential on deepwater temperature and strength of stratification due to its control of vertical light and heat distribution throughout the water column^8,19–21^. Controls on deepwater temperature and vertical thermal structure can also be moderated by lake morphology due to influences of fetch, basin shape, and depth^5,22^, which can also moderate the influence of other drivers on lake thermal structure, such as has been observed for the interaction between lake size and water clarity^23^. The interactions between stratification, wind stress, and basin morphometry also determine whether incoming heat is retained in the epilimnion or mixed to deeper depths^24^. Hence, drivers of, and changes in, full vertical thermal structure do not necessarily mimic those commonly reported for surface water temperatures, but are important if we are to understand the breadth of ecological consequences associated with changing lake thermal structure.

**Fig. 1:**
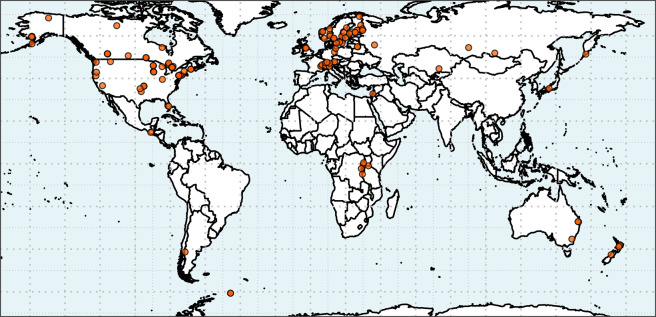
Map showing locations of the 153 lakes with vertical temperature profile data in this data set.

**Fig. 2:**
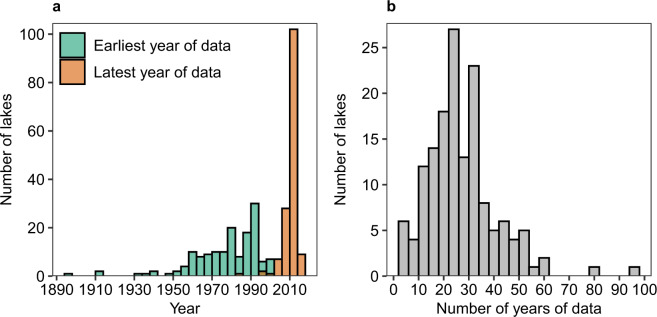
Histograms of temporal coverage for each lake in this data set. (**a**) Histogram of the earliest year (green) and latest year (orange) with temperature profile data for each lake. (**b**) Histogram of the total number of years with temperature profile data for each lake.

We present here a globally-expansive data set of vertical summertime temperature profiles of 153 lakes spanning 26 countries across all 7 continents (Fig. 1). Start and end years vary by lake, with starting years ranging from 1894 to 2002 and ending years ranging from 1986 to 2016 (Fig. 2). The median number of years with summertime vertical temperature profiles is 25 years, with a range from 3 to 96 years of data depending on the lake (Fig. 2). Lake characteristic data are also provided, including geographic, morphometric, and water quality variables (Online-only Table 1; Supplementary Fig. S1).

Our goal was to assemble and publish this globally-expansive data set of lake vertical temperature profiles to expand on prior global data sets of lake surface water temperatures^25^, and increase the understanding of changes in deepwater temperatures and the full vertical thermal structure of lakes over time. A collaborative working group at the Global Lake Ecological Observatory Network (GLEON; www.gleon.org) sought to analyze changes in lake thermal structure at a global scale by collecting long-term vertical temperature profile data from a broad range of lake types in order to address key scientific questions such as: How is vertical lake thermal structure changing over time and depth? What types of lakes are experiencing the most rapid changes in temperature at depth? How do trends in temperature at various depths or other metrics of thermal structure (i.e., mixing depth, strength of stratification) vary among lakes? Through this data set, questions such as these can be more fully addressed. This data set can be paired with meteorological or other climate or environmental data to analyze the drivers of vertical thermal structure in comparison with the established drivers of surface water temperatures, and to assess potential ecological consequences in times of global change. In addition, the data set can be used to calibrate and evaluate lake models, such as the Lake Model Intercomparison Project^26^ (LakeMIP) and the Inter-Sectoral Impact Model Intercomparison project^27^ (ISIMIP).

## Methods

Temperature profiles, sampling methods, and physical descriptions were compiled for 153 globally-distributed lakes. We selected vertical temperature profiles measured in the same location for multiple years, typically the deepest part of the lake, using a single mid-summer profile for each lake and year. Data are presented for all years for which profiles were available, with both raw vertical temperature profiles and interpolated vertical temperature profiles presented. Depth increments for raw profiles vary by lake based on sampling methodology, as described for each lake below. For interpolation, the selected temperature profiles were linearly interpolated or binned to 0.5 m increments throughout the water column. Mid-summer profiles for each lake were selected as described in (7). Briefly, we calculated relative thermal resistance to mixing^28,29^ (RTR) for all profiles for each lake for each year. The day of year with the maximum RTR value for each year was selected for each lake, and the median day of year across all years per lake was considered the target day for single mid-summer profile selection. For each lake and year, the temperature profile nearest to this median day of year ± 21 days was selected. If no profile spanning surface to near the maximum lake depth was available, then no profile was selected. Details on sampling methods from each contributing research group follows, organized by major geographic region and alphabetized within geographic region. Additional details for water chemistry sampling methods can be found in the Supplementary Information (Supplementary Table S1).

### Western North America

Castle Lake in California, USA has been sampled annually since 1959. Temperature data were measured between 12:00 and 14:00 at the deepest part of the lake in 1 m depth intervals. Measurements were taken using a reversing thermometer (1959–1975), Hydrolab (1975–1982; 2011–2013), and Yellow Springs Instruments (YSI) model 85 (1999–2011) (sampling instrument is unknown for most of the 1980s and 90s).

Crater Lake is located in the northwestern USA at the crest of the Cascade Mountains in Oregon. Temperature data were collected between 8:00 and 17:00 from the middle of the lake in the deepest basin. From 1988 to present, temperature profiles were measured by lowering a Seabird Instruments SBE19 CTD through the water column at a rate of approximately 0.5 m per second, collecting two readings per second. Data were binned to 1 m increments. Prior to 1988, temperature profiles were measured manually with a Montedoro-Whitney thermistor with a 250 m cable.

Emerald Lake, California, USA has temperature profiles measured from the deepest part of the lake beginning in 1984. Temperature was measured in 1 m increments throughout the water column from 1984 to 2006 using a YSI 58, after which a thermistor chain was deployed with Onset Water Temp Pro spaced at 0.5 to 1 m intervals.

Flathead Lake in northwest Montana, USA is the largest freshwater lake west of the Mississippi River by surface area. Temperature data were collected fortnightly in June, July, and August 1978–2013 with various instruments. From 1978–1984, data were collected with a Hydrolab I; from 1985–1993, with a Hydrolab Surveyor II; from 1993–2003, with a Hydrolab Surveyor III; and from 2003–2001, with a Hydrolab Surveyor IV. Since 2011, data have been collected with a Hydrolab DS5 unit. Measurements were taken between 10:00 and 14:00 at the deepest point of the lake in 1 to 10 m depth intervals throughout the water column, with greater intervals between measurements at deeper depths. The intervals between depths on a given sampling date also changed periodically early in the data set before consistent intervals were established. Instrumentation was calibrated before each sampling event to account for the elevation of the sampling site and highly variable barometric pressure.

Washington Lake is located in Washington, USA. Temperature profiles were measured between 9:00 and 16:00 in the main trench of the lake. Temperature was measured using various bathythermographs between 1933–1986, a Kahl digital temperature meter 202WA510 beginning in 1974, and a YSI 6600 V2 sonde beginning in 2012. For measurements with the bathythermographs, temperature was measured every 1 m close to the thermocline and every 5 m elsewhere. When the Kahl temperature meter was used, temperature was recorded for every meter through 20 m and in 5 m increments after 20 m depth. Data from the YSI sonde were recorded continuously, so temperature readings were used at similar intervals to those from the previous instruments.

### Central North America

Acton Lake is a eutrophic reservoir in southeastern Ohio, USA. Temperature was measured in the deepest part of the lake at 0.5 m depth increments from 0 to 5 m and 1 m increments for the remainder of the water column. Temperature data were typically collected in the mid-morning and occasionally in the early afternoon in mid-July of each year from 1992–2012 using a handheld YSI temperature/dissolved oxygen sensor or a YSI sonde.

Lakes Bighorn, Harrison, Pipit, and Snowflake are located along the eastern front range of the Rocky Mountains in the Cascade Valley of Banff National Park (Alberta, Canada). Temperature data were collected in the deepest point of the lake in 1 m depth intervals between 10:00 and 15:00 using a MK II Thermistor (Flett Research Ltd, Winnipeg, Canada) for measurements taken beginning in the 1990s. Earlier measurements (1960s and 1970s) were taken using a YSI Model 425 C thermistor thermometer, which was calibrated against a mercury thermometer.

Douglas Lake is a mildly eutrophic glacially formed multiple ice-block kettle lake in northwestern Cheboygan County, Michigan, USA. Temperature data were collected from 1913–2014 at 1 m intervals from 0 to 24 m in the center of South Fishtail Bay Kettle with a reversing thermometer at approximately 1 m increments (1913–1970), a YSI model 54 A oxygen electrode-thermistor thermometer (1971–1982), a Hydrolab MS-5 multiprobe (1983–2009), and an 8-node MHL thermistor string (2010–2014).

Lakes Eucha, Grand Lake O’ the Cherokees, Spavinaw, Texoma, and Thunderbird are reservoirs located in Oklahoma, USA. Temperature profiles were measured at the deepest point in these reservoirs between morning and mid-day with various sensors. YSI temperature probes were used for all samples collected in Lake Thunderbird, through 1991 in Grand Lake O’ the Cherokees, through 1995 in Lakes Eucha and Spavinaw, and through 2000 in Lake Texoma. Beginning in 2000 in Lake Texoma, Hydrolabs were used for temperature measurements, with a Hydrolab H2O through 2008, and Hydrolab DSX5 thereafter. A Hydrolab was used for measurements in Grand Lake O’ the Cherokees from 2011–2013. Lakes Eucha and Spavinaw were sampled with a Hydrolab H2O through 2005, a YSI 6930 V2 for samples from 2006–2012, and a YSI EXO1 for samples after 2012.

Katepwa Lake is a eutrophic, riverine site located in the Qu’Appelle River drainage basin in southern Saskatchewan, Canada. The lake has been sampled since 1994 as part of the Qu’Appelle Long-term Ecological Research network (QU-LTER). Temperature data were collected from a standard deep site in 1 m intervals between 10:30 and 13:00 using a YSI 85 or similar multi-parameter probe.

### Northeastern North America

Temperature profiles were collected beginning in 1969 for six lakes (Lakes 222, 224, 239, 240, 373, and 442) at the IISD Experimental Lakes Area (International Institute for Sustainable Development, Northwestern Ontario, Canada). Profiles were measured in the deepest part of the lake in 1 m intervals, except in the thermocline where temperature was measured every 0.25 m. Montedoro-Whitney thermistors (models TC-5A and TC-5C) were used through 1983, a Flett Research Mark II digital telethermometer was used for temperature measurements from 1984–2009, and a RBR XRX620 multifunction probe with integrated temperature sensor for measurements beginning in 2010.

The Dorset “A lakes”, Blue Chalk, Chub, Crosson, Dickie, Harp, Heney, Plastic, and Red Chalk Lakes are located in the Muskoka-Haliburton region of south-central Ontario, Canada. The study sites are primarily small headwater lakes, with the exception of Red Chalk Lake which is located downstream of Blue Chalk Lake. Temperature data were collected from the deepest point in each lake using a YSI 58 temperature/dissolved oxygen meter (or occasionally a digital YSI 95 meter) beginning in the late 1970s. Measurements were collected between 9:00 and 16:00, with readings taken every 1 m from the lake surface (0.1 m depth) to within approximately 1 m of lake sediments.

Bubble Pond, Eagle Lake, and Jordan Pond are located on Mount Desert Island off the coast of Maine, USA. Temperature data were collected from the location of the maximum depth at 1 m increments using a YSI 600XL multiparameter water-quality monitor (sonde) from 2006 to present and a YSI 54ARC before this time.

Lake Champlain is located in the northeastern USA, on the border of Vermont and New York state and partly extending into Quebéc, Canada. Temperature data were collected from a sampling station in Mallet’s Bay beginning in 1992. Measurements of temperature were taken at 1 m intervals between 8:00 and 17:00 using a Hydrolab MS-5 multi-probe sonde.

Clearwater, Sans Chambre, and Whitepine Lakes are located in northeastern Ontario, Canada, and Hawley Lake is in the Hudson Bay Lowlands area of subarctic Ontario, Canada. Temperature profiles were measured from near the area of maximum depth, beginning in the 1970s in Clearwater and Hawley, and beginning in the 1980s in Sans Chambre and Whitepine. Measurements were taken in 1 m intervals through the water column between 12:00 and 17:00. Clearwater, Sans Chambre, and Whitepine Lakes’ temperature profiles were measured using various YSI temperature/dissolved oxygen meters (models 50B, 51B, 52, 54, or 58) beginning in 1998, with a YSI model 54 temperature/dissolved oxygen meter used for Sans Chambre and Whitepine for most earlier years’ measurements. In Clearwater Lake, a YSI model 432D telethermometer was used through 1975, a Montedoro-Whitney TC-5C thermistor from 1976–1981, and a Mark II Telethermometer from 1982–1998. In Hawley Lake, various YSI temperature probes were used in earlier years, and since 2009, a YSI Pro ODO meter was used to measure water temperature.

Lakes Giles and Lacawac are located in the Pocono Mountains region of Pennsylvania, USA. Temperature data were collected from the deepest point in the lake in late July or early August each year beginning in 1988, between 9:00 and 16:00. Temperature measurements were measured in 1 m increments using a YSI 58 temperature/dissolved oxygen meter (1988–1992), or with a rapid recording Biospherical Instruments PUV 500 (1993–2003) or BIC 2104 P (2004-present) recording at 4 Hz while being lowered through the water column. Profiles taken with the YSI were linearly interpolated to 0.5 m depth increments, and profiles taken with the PUV and BIC were binned to 0.5 m depth increments.

Lake Lillinonah is a reservoir on the Housatonic River located in western Connecticut, USA. Temperature data were collected at the deepest point of the lake between 9:00 and 17:00 beginning in 1996 by First Light Power. Measurements were collected every 5 m using a YSI 58 temperature/dissolved oxygen meter.

Mohonk Lake is a small glacial lake with a single deep basin located at the Shawangunk Ridge, New York, USA. Temperature data were collected from the northern end of the lake from 1984–2013 during daytime. Temperature measurements were measured manually in 1 m increments using Digi-sense Economical Thermistor 400 series (Model #93210-00). Additional weekly temperature profiles are publicly available^30^.

Temperature profiles were compiled from 11 lakes from the North Temperate Long Term Ecological Research Network in Wisconsin, USA. Fish, Mendota, Monona, and Wingra Lakes are located in southern Wisconsin, and Allequash, Big Muskellunge, Crystal Bog, Crystal Lake, Sparkling, Trout Bog, and Trout Lake are in northern Wisconsin. Temperature profiles were measured in 1 m increments from the surface to lake bottom at the deepest location in each lake since 1981 (northern lakes) and 1995–1996 (southern lakes), and since 1894 in Mendota^31^. Various temperature/dissolved oxygen probes were used to collect these data, and were calibrated in the field prior to data collection. For the northern lakes, a Montedoro Whitney CTU-3B sensor was used for some data collected between 1981–1986, a Whitney TC-5C for 1982–1983, Whitney DOR-2A in 1984, YSI-57 in 1983 and 1985, and a YSI-58 for 1985 onward. Temperature data in the southern lakes were collected with a YSI-58 temperature/dissolved oxygen sensor.

Lake Opeongo is the largest oligotrophic deepwater lake in Algonquin Provincial Park, Ontario, Canada. Temperature data were collected by Ontario Ministry of Natural Resources and Forestry staff at Harkness Fisheries Research Station in the west basin of Opeongo’s South Arm in midsummer in 1958–1965, and between 9:00 and 16:00 in 1998–2014. Temperature loggers (Hobo Tidbits, 1998–2004; Onset Water Temperature Pro V1, 2004–2008; and Onset Pro V2, 2009–2014) were installed after ice out in the same location at approximately 1.5 m intervals to a 15 m depth, with a deepwater thermistor placed at approximately 20 m. Temperatures were recorded at high temporal resolution (10 to 15 minute intervals) throughout the summer using these temperature strings. Earlier (1958–1965) temperature profiles were measured using handheld thermistors.

Lake Sunapee is the fifth largest lake located within New Hampshire, USA. Temperature data were collected in the morning from the deepest point of the lake in the central basin beginning in 1986. Temperature measurements were measured manually in 1 m increments using a YSI 52 temperature meter.

Lake Wallenpaupack is a reservoir in the Pocono Mountains region of Pennsylvania, USA. Temperature profiles were measured in the center of the lake during daytime hours, with measurements taken every 0.5 to 1 m. Temperature was measured with various YSI instruments including a YSI 610-DM/600XL (2002–2005), YSI 85 (2008–2010), YSI 600XL sonde with 600D datalogger (2011–2012), and various temperature sensors prior to 2002.

### Southeastern North America

Lake Annie is located in the Lake Wales Ridge region, Florida, USA. Temperature data were collected from the deepest point in the lake on a monthly basis between 9:00 and 16:00 beginning in 1984. Temperature measurements were measured manually in 1 m increments using a Montedoro Corporation Thermistor Model TC-5c (1984–2008) and a YSI Pro Plus (2009–2014) with values measured while lowering the meter to the bottom of the lake and again when raising it to the surface. The data reported are the means of the two depth-specific values.

Temperature data were measured in Lake Okeechobee in Florida, USA beginning in 1973. Temperature data were measured near the surface at approximately 0.2 m between 8:00 and 12:00 using a Hydrolab through 1995 and a YSI 58 temperature sensor from 1996–2014.

### Subarctic

Aleknagik, Beverley, Chignik, Hidden, Kulik, Little Togiak, Lynx, and Nerka Lakes are located in Alaska, USA. Temperature profiles have been measured on these lakes since the 1960s (Aleknagik, Beverley, Kulik, Little Togiak, and Nerka Lakes), and since the early 2000s (Chignik, Hidden, and Lynx Lakes). Measurements were taken during the day, typically between 10:00 and 19:00. A bathythermograph was used for temperature measurements through 1967, a digital thermistor from 1968–1998, a YSI 660 sonde for samples from 1999–2012, and a YSI Castaway beginning in 2013, with the exception of samples from Chignik Lake, for which a handheld thermometer was used to measure the water temperature from Van Dorn casts.

Toolik Lake is a kettle lake located in Alaska, USA. Temperature data were collected between June and August in the south basin of the lake between 9:00 and 11:00. Temperature was recorded in 1 m increments throughout the water column with a Hydrolab profiler sampling Surveyor 4a datalogger and a datasonde 4a multiprobe^32–35^.

Vulture Lake is located in the sub-Arctic region of the Northwest Territories, Canada. Temperature data were collected from one of the deeper parts of the lake in late July or early August during the open-water season from 1997–2014. Temperature measurements were recorded using a multi-probe sonde (e.g., YSI 6820) at 0.5 m or 1 m increments (every 1 m for most years, except 1999 [every 0.5 m] and 2009 [every 0.2 m]). The corresponding depth was measured using the readings from the depth sensor. Measurements were collected continuously from just below the lake’s surface to approximately 0.5 m above the water-sediment interface. Sensors equipped on profiling instruments were calibrated following the manufacturer’s recommended frequency and methods to ensure accurate and reliable operation of the sensors in the field.

### Central and South America

Lake Atitlán is a deep tropical mountain lake in the Guatemalan highlands, sampled in 1968–1969 and 2010–2011. Temperature profiles at the lake center were measured manually between 7:00 and 13:00 in variable increments (1 to 5 m) in the first 30 m using a YSI 51 or YSI 95 temperature/dissolved oxygen meter. For depths below 30 m, samples were collected using a Van Dorn bottle and temperature was measured immediately upon the sample reaching the surface.

Lake Mascardi is located in the North Patagonian Andes in Argentina. Temperature data were collected near the deepest point in the Catedral arm of the lake in mid-summer (January-February) between 12:00 and 14:00 beginning in 1994. Temperature measurements were taken using rapid recording Biospherical Instruments PUV 500 (1994) or PUV 500B (1996–2014) recording at 4 Hz while being lowered through the water column.

### Africa

Lake Kivu is located on the border between Rwanda and the Democratic Republic of Congo, and is one of the seven African Great Lakes. Temperature data were collected in the Ishungu basin beginning in 2002 between 9:00 and 16:00. Temperature was measured in 5 m increments using a YSI 55 temperature/dissolved oxygen meter (2002–2005), or with a suite of instruments (YSI 6600 V2, Hydrolab DS4a, DataSonde 4a 42071, Sea and Sun 725 and 257) recording at high frequency while being lowered through the water column. All temperature profiles were vertically interpolated to a regular vertical grid with 1 m increments using piecewise cubic Hermite interpolation^18,26^.

Lake Nkugute is located in Uganda. Temperature data for Lake Nkugute were collected in the deepest part of the lake at a depth interval of 1 to 5 m using a liquid-in-glass thermometer set in a Ruttner sampler in 1964 with the contemporary (2002-present) data being measured with a YSI sonde.

Lake Nkuruba is located in western Uganda, in the vicinity of Kibale National Park (northern sector). Temperature data were collected beginning in 1992 from the deepest part of the lake in 1 m increments through a depth of approximately 30 m. Temperature was measured manually using a YSI 50 or 51B temperature/dissolved oxygen meter.

Lake Tanganyika has temperature profile data dating back to 1912. Temperature profiles in this lake were typically measured in the morning, between 9:00 and 12:00 from the north basin of the lake near Kigoma, Tanzania. Temperature profiles over this century of data collection have been taken using a multitude of instruments. Since 1993, temperature profiles have been measured using a YSI 6600 V2 sonde, titanium RBRduo TD, Seacat Profiler V3.1b, Onset HOBO U22 temperature loggers, CTD Seabird 19, STD-12 Plus CTP profiler, and a YSI 58 temperature/dissolved oxygen meter. Prior to 1993 various methods were used, including measurement of water temperature from Van Dorn casts with a mercury thermometer, various data loggers, reversing thermometers, and Niskin bottles and a bathythermograph^36^.

Lake Victoria is shared between Tanzania, Uganda, and Kenya. Temperature data were collected from stations across the lake during acoustic surveys each year in 2000–2001 and in 2008 using a submersible Conductivity Temperature-Depth profiling system (CTD, Sea-bird Electronics, Sea Cat SBE 19).

### Scandinavia and Northern Europe

Lakes Allgjuttern, Brunnsjön, Fiolen, Fracksjön, Övre Skärjön, Remmarsjön, Rotehogstjärnen, St. Skärsjön, Stensjön, and Stora Envättern are relatively small, boreal lakes in Sweden. In contrast, Lake Vänern is the largest lake in Sweden. Temperature data have been collected since 1988 (since 1973 for Lake Vänern) between morning and mid-afternoon. Temperature measurements were taken from the deepest point in each lake from the surface through 1 m above the lake bottom at depth intervals varying between 1 m and up to 10 m (in Lake Vänern).

Lakes Byglandsfjorden, Hornindalsvatnet, Mjøsa, Øyeren, Selbusjøen, and Strynevatnet are all large and deep lakes located in the central region of western Norway. Temperature profiles were measured in the deepest part of the lake between 9:00 and 16:00 beginning in the mid-1990s. Temperature was measured using an Aanderaa 4060 every meter in the upper part of the water column, with greater than 1 m intervals between measurements in depths below 20 m. For the past 2 to 3 years of data collection, a Castaway CTD recorded temperatures while lowered. Data collection was made by The Norwegian Water Resources and Energy Directorate (NVE).

Temperature data were collected from Sweden’s Lake Erken from the deepest point in the lake in 1 m intervals between 7:30 and 9:30 beginning in 1940. Temperature was measured at 1 m intervals using a variety of instruments. In early years, a thermometer inside a transparent Ruttner sampler was read to obtain the temperature of water collected from different depths. Later, underwater thermistors were used from a variety of manufacturers. In recent years, combined temperature and dissolved oxygen sensors have been used to collect water temperature measurements: a YSI model 52 (1996–2006), WTW Oxi 340i (2006–2012), and Hach HQ40d sensor system (2012-present).

Lakes Inarijärvi, Kallavesi, Konnevesi, Näsijärvi, Päijänne, Pielinen, and Pyhäjärvi are generally large lakes located throughout Finland, from southern Finland to the northern-most part of the country (Lapland). Lake Pesiöjärvi in the same region has a significantly smaller surface area than others. Lakes Konnevesi and Päijänne have two different temperature profile sites (Konnevesi: Näreselkä and Pynnölänniemi, Päijänne: Linnasaari and Päijätsalo). Temperature data were typically collected in the deepest part of the lake, or in case of large fragmented lakes, the deepest part of the respective basin. Reversing mercury thermometers (with or without Ruttner water samplers) were used until the digital temperature measurements were introduced in the early 1970s. HL Hydrolab Ab PT77A (approximately 1975–1995), DeltaOhm HD8601P (1995–2005), and HT Hydrotechnik Type 110 (2005–2014) have been used for measuring water temperature profiles. However, all device types have been used at each station as long as they have worked. Unfortunately, site-specific documentation of devices used during different years are not available. Before the 1980s, measurements were collected every 5 m before and every 10 m after a depth of 20 m. Since 1980–1981, measurements have been made in 1 m intervals from the surface through 20 m, every 2 m from 20 to 50 m, and every 5 m past 50 m.

Lake Pyhäselkä (Pyhaselka) is a large, humic lake located in North Karelia, Finland. It is the northernmost basin of the Saimaa lake system. Temperature data were collected from the deepest point in the lake (Kokonluoto) in 5 m depth intervals beginning in 1962 between 8:00 and 16:00 using a thermometer in a Ruttner water sampler.

### Central Europe

Lakes Annecy, Bourget, and Geneva are located in eastern France^37^. Temperature data were collected from the deepest point in the lake between 9:30 and 11:00, beginning in 1991 for Lake Annecy, in 1984 for Lake Bourget, and in 1974 for Lake Geneva. In Lake Annecy, various multiparameter probes, including Meerestechnik Elektronik (1991–2001), CTD 90 (2003–2005), CTD 90 M (2008–2011, 2013), and RBR (2012) were used for temperature measurements at depth intervals between 0.01 and 1.8 m. Temperature profiles were measured in Lake Bourget at depth intervals between 0.01 and 10 m using various multiparameter probes, including ISMA probe DNTC (1984–1985), Meerestechnik Elektronik ECO 236 (1986–1998), CTD SBE 19SeaCAT Profiler (1992–2002), and CTD SBE 19plus V2 SeaCAT (2003–2013). In Lake Geneva, water temperature was measured from water samples with a thermometer until 1990 from discrete depths of 5 to 10 m intervals through 50 m depth and approximately 50 m intervals for the rest of the water column. After 1990 various multiparameter probes were used for temperature measurements, including Meerestechnik Elektronik (1991–2001), CTD 90 (2002–2007), CTD 90 M (2008–2011, 2013), and RBR (2012). Data for Annecy, Bourget, and Geneva are available^38^ at https://data.inrae.fr/dataset.xhtml?persistentId=doi:10.15454/YOLA0Y.

The Cumbrian lakes in the English Lake District, Bassenthwaite Lake, Blelham Tarn, Derwent Water, Esthwaite Water, Grasmere, and Windermere North Basin, are located in northwest England. Temperature data were collected in midsummer at the deepest point of each between 9:00 and 14:00 beginning in 1991. Temperature profiles were measured at depth intervals judged in the field depending on the stratification pattern and depth of the lake. Temperature was measured with various combined temperature/oxygen sensors, including a YSI 58 (1991–2002) and WTW Oxi 340 (2002–2013) in Windermere North Basin, and a YSI 58 (1991–2002), WTW Oxi 340i (2002–2010), and Hach HQ 30d and LDO probe (2010–2013) in all other lakes.

Lake Constance is located on the border of Germany, Switzerland, and Austria. Temperature profiles were measured at the deepest part of the central basin (Upper Lake Constance) of the lake beginning in 1964, with measurements taken between 9:00 and 10:00 using a thermometer. Depth intervals between samples increased with depth, with measurements taken every 2.5 to 5 m through 20 m, every 10 m through 50 m, and every 50 m down to a depth of 250 m^39^.

Lake Mondsee is located in the Lake District “Salzkammergut” of Austria. Temperature data were collected from the deepest point of the lake approximately monthly near noon beginning in 1968. Temperature measurements were usually measured at depth intervals of 1 to 2 m in the epilimnion and 5 to 10 m intervals in the hypolimnion using a thermometer housed in a Schindler sampler prior to 1998. Beginning in 1998, data were extracted from continuous YSI 6920 profiler readings, with a YSI 6600 used beginning in 2008 and a thermistor chain from 2010–2013.

Lake Müggelsee, located in Berlin, Germany was sampled weekly beginning in 1978 between 8:00 and 9:00. Temperature was measured every 0.5 to 1 m at the deepest part of the lake using a Hydrolab H2O sensor beginning in 1992, and a thermistor probe for years prior to 1992.

Lake Piburgersee is located in Tyrol, Austria. Temperature was measured using a calibrated thermometer every 3 m throughout the water column in the deepest part of the lake, beginning in 1970. Additional information and analyses are available elsewhere^40^.

Plusssee (Plußsee) is located in northern Germany (Schleswig-Holstein). Temperature data were collected from the deepest point of the lake between 9:00 and 15:00 beginning in 1971. Temperature was measured manually in 1 m increments from 0 to 15 m and in 5 m increments from 15 to 25 m using a thermometer mounted into a Ruttner sampler prior to 1976, and after 1976 with a WTW temperature/dissolved oxygen probe.

Traunsee is a large and deep oligotrophic lake in the Salzkammergut lake district of Austria. Temperature data were collected at the deepest point of the lake between 9:00 and 12:00 beginning in 1965. Temperature was measured at 2 to 5 m intervals through 20 m, and at 20 m intervals through the rest of the water column using a mercury thermometer mounted in a 5-liter water sampler.

Lower Lake Zurich is located in Switzerland. Temperature profiles were measured at the deepest part of this lake between 8:30 and 12:00 beginning in 1936. Measurements were taken at the deepest point in the lake using a range of sensors, mainly NTC thermistors (1936–2000). Beginning in 2001 various sondes were used, including FLP-10 multisonde (2001–2008), multisonde Hydrolab DS5 (2008–2015) at 0.5 to 1 m intervals through 30 m, 5 m intervals through 50 m, and 10 m intervals throughout the rest of the water column.

### Southern Europe

Lake Garda is one of the largest lakes in Europe, and the largest Italian lake. Owing to its deep depth, Lake Garda is characterized by long periods of incomplete vertical winter water circulation, which are interrupted by full mixing of the water column after the occurrence of harsh winters. Limnological investigations have been carried out since 1991 in a pelagic station located at the point of maximum depth of the northwest basin. Profiles of water temperature were recorded during the summer months using Idronaut Ocean Seven 401 (1991–1997), Seacat SBE 19–03 (1998–2008), and Idronaut Ocean Seven 316Plus since 2009^25^.

Lake Iseo is located in northern Italy (Lombardy Region). It is a deep mesotrophic lake, characterized by long periods of incomplete vertical winter water circulation^41^. Temperature was measured at the deepest point in the lake using an automatic thermistor probe coupled with an oxygen sensor from 1993 to 2011 with Microprocessor Oximeter WTW OXI 320 and from 2012 to 2016 with Microprocessor WTW multi 3410. Temperature was measured for at least ten discrete depths and all measurements were regularly checked with a mercury-filled Celsius reversing thermometer.

Lake Lugano is located in the foothills of the Central Alps, on the border between Switzerland and Italy. The lake is divided into a northern and southern basin, which are separated by a causeway (built on a natural moraine). Due to reduced connectivity^42^ (flow of approximately 0.38 km^3^ year^−1^ from north to south) and different morphometric characteristics, the two basins were considered separately in the data set. Temperature profiles were collected at sites near the deepest point of each basin. Temperature was measured using reversing thermometers from 1974–1979 and multiparameter probes thereafter (Hydropolyester HTP 77 during 1980–1985, Ocean Seven 401 during 1986–1993, Ocean Seven 316 during 1994–2015). As an exception, temperatures at depths greater than 100 m were also measured using a reversing thermometer between 1980–1985. Temperature was measured for at least nine or seven discrete depths for the northern and southern basins, respectively, from 1974–1986, whereas full temperature profiles with vertical resolution of 0.5 to 1 m were measured between 1987–2015.

Lake Maggiore is a deep lake located in northwestern Italy and Switzerland, south of the Alps. Lake Maggiore can be classified as holo-oligomictic, with complete overturns only occurring at the end of particularly cold and windy winters. Temperature data have been collected at the deepest point of the lake since 1981, usually between 10:00 and 12:00 at the Ghiffa station. Temperature has been measured at discrete depths of 0, 5, 10, 20, 30, 50 m, and every 50 m through 360 m using mercury-filled thermometers connected to the bottle used for water sampling^43^.

### Eastern Europe

Lakes Batorino, Myastro, and Naroch are located in the northwest part of Belarus, in the glacial landscape. Temperature data were collected monthly in the center of the lake during the vegetative season of May to October beginning in the 1950s and 60s. Measurements of water temperature were taken every 2 to 4 m through the water column between 9:00 and 14:00 with a mercury deepwater thermometer with a scale resolution of 0.1°C mounted in a metal frame, or in a Ruttner sampler.

### Russia

Lake Baikal is located in Siberia, Russia. Temperature data were collected from a station situated 2.8 km from the shoreline near the Bolshie Koty settlement at depths of 0 and 50 m between 9:00 and 12:00 from 1948–2009. Temperature was measured with a mercury thermometer inside a Van Dorn bottle.

Lake Glubokoe is located in Central European Russia, Moscow Province. Temperature profile data were collected from the deepest point of the lake from the surface through 10 m. Since 1982, water was taken from the required depth with a 10 L bathometer and its temperature was measured with a mercury thermometer; instrumentation prior to 1982 is unknown.

Kurilskoye Lake in Kamchatka, Russia was sampled beginning in 1942. Temperature profiles were measured in the deepest part of the lake between 8:00 and 15:00. Various temperature sensors were used over time, including a reversing thermometer (1942–1965), bathythermograph (1980–2003), Hydrolab (2004–2008), and RINKO profiler (2009–2014).

Lake Shira is located in the south of Siberia, Russia. Temperature profiles were measured in the deepest part of the lake between 11:00 and 15:00 from the surface to the depth of 20 to 24 m with various temperature sensors including multisonde Hydrolab 4 A (2000–2008) and a YSI 6600 (2009–2014).

### Middle East

Lake Kinneret is located in Israel (Jordan Valley). Temperature data were collected near the deepest point of the lake (Station A) between 7:00 and 16:00 from 1969–2013, measured every centimeter with an error of ± 0.005 °C, and averaged to every 1 m. Temperature measurements were taken from 1969 to 1986 using an underwater thermometer (Whitney-Montedoro), from 1987 to 2003 using a STD-12 Plus (Applied Microsystems), and from 2003 to 2013 using AML Oceanographic Minos•X.

### Asia

Lake Biwa is located in the central part of the Japanese archipelago (Shiga Prefecture, Japan). Temperature data were collected from a station near the deepest part of the lake from 1958–2010. Temperature was mostly measured between 9:00 and 12:00 at 5 m intervals from 1959–2005, and at 1 m intervals beginning in 2006. Measurements were made using an electronic thermometer (Murayama Denki Ltd.) from 1958–1970, a thermistor thermometer (Shibaura electronics, HCB III) from 1970–1994, a CTD profiler (Alec electronics, ABT-1) from 1994–2006, and a CTD profiler (JFE Advantech, compact-CTD) from 2007–2010.

### Australia

Lake Burley Griffin is a reservoir constructed in 1963 by damming the Molonglo River. It is located in the geographic center of Canberra, the capital of Australia. Temperature data were collected from the deepest point of the reservoir, near the dam wall. Profiles were measured in 1 m depth intervals (reduced to 3 m intervals in 1992) between 8:45 and 16:15 from 1981–2010 by the National Capital Authority.

Lakes Samsonvale (North Pine) and Somerset are located on the east coast of Australia, in southeast Queensland. Temperature in each lake was measured at a site approximately 100 m from the dam wall. Samsonvale’s (North Pine’s) temperature was measured using a YSI 6560 sensor on a YSI 6600 V2 sonde beginning in 2009 continuously over a 24-hour period at 1 m intervals. Prior to 2009, temperature was measured via a thermistor string with various unknown instruments. Somerset’s temperature profiles from 2000–2002 were measured using temperature sensors on a thermistor string, spaced at 0.5 m intervals through 3 m, 1 m intervals through 7 m, 2 m intervals through 17 m, and 3 m intervals for the rest of the water column.

### New Zealand

Lakes Brunner and Taupo are located in New Zealand, in the West Coast and Waikato regions, respectively. Temperature profiles were measured at the deepest part of these lakes between 9:00 and 16:00, beginning in the early 1990s. Temperature was measured by lowering CTD profilers through the water column, using an YSI EXO sonde (Brunner) and RBR profiler (Taupo).

Lakes Okareka, Okaro, Okataina, Rerewhakaaitu, Rotoehu, Rotoiti, Rotoma, Rotorua, Tarawera, and Tikitapu are located in Rotorua, Bay of Plenty, New Zealand. Temperature profiles were measured between 10:00 and 14:00 in the central basin using Seatech CTD casts with a Seabird 19Plus or 19PlusV2 beginning in 2003. Temperature was measured at a frequency of 4 Hz during each cast and data were binned to 1 m depth intervals. Profiles before 2003 were measured in 1 m depth intervals with either a YSI Water Quality Logger 3800 or YSI Sonde model 3815.

### Antarctica

Lakes Heywood (1962–1995), Moss (1972–2003), and Sombre (1973–2003) are located in the South Orkney Islands, Antarctica. Temperature data from these lakes were collected using a Mackareth-type probe at 1 to 2 m intervals in the deepest part of the lake.

## Data Records

Data are presented in three comma delimited files^44^. The first contains the pertinent metadata for each lake (“SiteInformation.csv”), including information about the source and contacts for each lake, sampling details, and the geographic, morphometric, and water quality characteristics (Online-only Table 1). The second two files contain the raw (“TempProfiles_Raw.csv”) and interpolated vertical temperature profiles (“TempProfiles_Interpolated.csv”) from each summer for each lake. These three files can all be linked with the LakeID column present in each file. All files are available at the Environmental Data Initiative, accessible at https://portal.edirepository.org/nis/mapbrowse?scope=edi&identifier=705. Additional information, details, and methods can be acquired by contacting the data providers at the emails found in “SiteInformation.csv”.

## Technical Validation

Quality control and assurance of temperature profile data was completed iteratively at multiple steps to ensure quality of all data. Both raw and interpolated data were visually inspected using contour maps to assess data accuracy over depth and time. At this stage, any data points that appeared inaccurate were removed, and then linear interpolation was re-run to fill in the missing data gaps. We used the same process to visually check vertical temperature data over depth and time following selection of single summer profiles for each lake. We also visually inspected time series plots of surface and deepwater temperature for each lake over time as an additional quality check. Any suspect vertical temperature profiles were removed, and, when possible, replaced with another temperature profile from the same lake with a similar sampling date.

## Supplementary information

Supplementary Figure S1

Supplementary Table S1

## Data Availability

All data compilation and creation of figures were conducted in R version 4.0.2^45^. R code can be found at https://www.github.com/rmpilla/GlobalTempProfileData.

## Online-only Table

**Online-only Table 1:** Column names and descriptions of site metadata file (“SiteInformation.csv”).

Header	Description
SiteID	Identifying number given to each lake or set of lakes whose data were managed by the same group
LakeID	Identifying number given to each lake. This ID is unique to each lake, and can be used as a primary key to link the SiteInformation table to the data in the TempProfiles tables
LakeName	Name (most common) by which lake is known
AlternateLakeName	Alternate names by which lake is known (if relevant)
LakeOrReservoir	Defines if body of water is identified as a natural lake or human-made reservoir
CountryOfLake	Country in which lake can be found
Region	Geographical region in which lake can be found
Latitude	Latitude of lake/approximate sampling site
Longitude	Longitude of lake/approximate sampling site
Elevation_m	Elevation of lake above sea level in meters
SurfaceArea_km2	Surface area of lake in square kilometers
Volume_km3	Volume of lake in cubic kilometers
MaxDepth_m	Maximum depth of lake in meters
MeanDepth_m	Mean depth of lake in meters
Secchi_m	Average Secchi depth of lake in meters (representative of recent years)
Chlorophyll_ug_L	Average chlorophyll concentration of lake in micrograms per liter (representative of epilimnion/surface waters in recent years)
TotalPhosphorus_ug_L	Average total phosphorus concentration of lake in micrograms per liter (representative of epilimnion/surface waters in recent years)
DissolvedOrganicCarbon_mg_L	Average dissolved organic carbon concentration of lake in milligrams per liter (representative of epilimnion/surface waters in recent years)
Contributor	Name(s) of data set contributor(s). If more than one main data contributor, names are separated by semicolons
ContributorContact	Contact (e-mail) of data set contributor(s). If more than one main data contributor, e-mails are separated by semicolons
ContributorInstitution	Institution(s) with which contributor(s) are associated. If more than one main data contributor, institutions are separated by semicolons

## References

[1] Adrian R (2009). Lakes as sentinels of climate change. Limnol. Oceanogr..

[2] Williamson CE, Saros JE, Vincent WF, Smol JP (2009). Lakes and reservoirs as sentinels, integrators, and regulators of climate change. Limnol. Oceanogr..

[3] Schneider P, Hook SJ (2010). Space observations of inland water bodies show rapid surface warming since 1985. Geophys. Res. Lett..

[4] O’Reilly CM (2015). Rapid and highly variable warming of lake surface waters around the globe. Geophys. Res. Lett..

[5] Kraemer BM (2015). Morphometry and average temperature affect lake stratification responses to climate change. Geophys. Res. Lett..

[6] Richardson DC (2017). Transparency, geomorphology and mixing regime explain variability in trends in lake temperature and stratification across Northeastern North America (1975-2014). Water.

[7] Pilla RM (2020). Deeper waters are changing less consistently than surface waters in a global analysis of 102 lakes. Sci. Rep..

[8] Pilla RM (2018). Browning-related decreases in water transparency lead to long-term increases in surface water temperature and thermal stratification in two small lakes. J. Geophys. Res. Biogeo..

[9] Woolway RI, Merchant CJ (2019). Worldwide alteration of lake mixing regimes in response to climate change. Nat. Geosci..

[10] Foley B, Jones ID, Maberly SC, Rippey B (2012). Long-term changes in oxygen depletion in a small temperate lake: Effects of climate change and eutrophication. Freshwater Biol..

[11] Knoll LB (2018). Browning-related oxygen depletion in an oligotrophic lake. Inland Waters.

[12] Verburg P, Hecky RE, Kling H (2003). Ecological consequences of a century of warming in Lake Tanganyika. Science.

[13] De Stasio BT, Hill DK, Kleinhans JM, Nibbelink NP, Magnuson JJ (1996). Potential effects of global climate change on small north-temperate lakes: Physics, fish, and plankton. Limnol. Oceanogr..

[14] Cohen AS (2016). Climate warming reduces fish production and benthic habitat in Lake Tanganyika, one of the most biodiverse freshwater ecosystems. P. Natl. Acad. Sci..

[15] Hansen GJA, Read JS, Hansen JF, Winslow LA (2017). Projected shifts in fish species dominance in Wisconsin lakes under climate change. Glob. Change Biol..

[16] Schmid M, Köster O (2016). Excess warming of a Central European lake driven by solar brightening. Water Resour. Res..

[17] Woolway RI, Meinson P, Nõges P, Jones ID, Laas A (2017). Atmospheric stilling leads to prolonged thermal stratification in a large shallow polymictic lake. Climatic Change.

[18] Thiery W (2014). Understanding the performance of the FLake model over two African Great Lakes. Geosci. Model Dev..

[19] Gaiser EE, Bachmann R, Battoe L, Deyrup N, Swain H (2009). Effects of climate variability on transparency and thermal structure in subtropical, monomictic Lake Annie, Florida. Fundam. Appl. Limnol..

[20] Read JS, Rose KC (2013). Physical responses of small temperate lakes to variation in dissolved organic carbon concentrations. Limnol. Oceanogr..

[21] Rose KC, Winslow LA, Read JS, Hansen GJA (2016). Climate-induced warming of lakes can be either amplified or suppressed by trends in water clarity. Limnol. Oceanogr. Lett..

[22] Winslow LA, Read JS, Hansen GJA, Hanson PC (2015). Small lakes show muted climate change signal in deepwater temperatures. Geophys. Res. Lett..

[23] Fee EJ, Hecky RE, Kasian SEM, Cruikshank DR (1996). Effects of lake size, water clarity, and climatic variability on mixing depths in Canadian Shield lakes. Limnol. Oceanogr..

[24] MacIntyre S (2009). Climate related variations in mixing dynamics of an Alaskan arctic lake. Limnol. Oceanogr..

[25] Sharma S (2015). A global database of lake surface temperatures collected by *in situ* and satellite methods from 1985–2009. Sci. Data.

[26] Thiery W (2014). LakeMIP Kivu: Evaluating the representation of a large, deep tropical lake by a set of one-dimensional lake models. Tellus A: Dynamic Meteorology & Oceanography.

[27] Vanderkelen I (2020). Global heat uptake by inland waters. Geophys. Res. Lett..

[28] Wetzel, R. G. *Limnology: Lake and River Ecosystems*. (Academic Press, 2001).

[29] Kalff, J. *Limnology: Inland Water Ecosystems*. (Prentice Hall, 2002).

[30] Mohonk P (2020). Environmental Data Initiative.

[31] Robertson D (2016). Environmental Data Initiative.

[32] Giblin A, Kling G (2016). Environmental Data Initiative.

[33] Giblin A, Kling G (2016). Environmental Data Initiative.

[34] Giblin A, Kling G (2016). Environmental Data Initiative.

[35] Giblin A, Kling G (2019). Environmental Data Initiative.

[36] Craig H (1974). UC San Diego: Library—Scripps Digital Collection.

[37] Rimet, F. *et al*. The Observatory on LAkes (OLA) database: Sixty years of environmental data accessible to the public. *J. of Limnol*. 164-178 (2020).

[38] Pilla RM, Anneville O (2020). Portail Data INRAE, V1.

[39] Straile D, Jöhnk KD, Rossknecht H (2003). Complex effects of winter warming on the physicochemical characteristics of a deep lake. Limnol. Oceanogr..

[40] Niedrist GH, Psenner R, Sommaruga R (2018). Climate warming increases vertical and seasonal water temperature differences, and inter-annual variability in a mountain lake. Climatic Change.

[41] Leoni B (2019). Long-term studies for evaluating the impacts of natural and anthropic stressors on limnological features and the ecosystem quality of Lake Iseo. Adv. Oceanogr. Limnol..

[42] Barbieri A, Mosello R (1992). Chemistry and trophic evolution of Lake Lugano in relation to nutrient budget. Aquat. Sci..

[43] Ambrosetti W, Barbanti L (1999). Deep water warming in lakes: an indicator of climatic change. J. Limnol..

[44] Pilla RM (2021). Environmental Data Initiative.

[45] R Core Team. R: A Language and Environment for Statistical Computing. http://www.R-project.org/ (R Foundation for Statistical Computing, Vienna, 2020).

