# Supplementary material for: Global data set of long-term summertime vertical temperature profiles in 153 lakes
Source: Sci Data. 2021 Aug 4;8:200. doi: 10.1038/s41597-021-00983-y (PMC8339007; doi:10.1038/s41597-021-00983-y)
Supplement: Supplementary file 1 — Supplementary Figure S1 [file 41597_2021_983_MOESM1_ESM.docx]

###
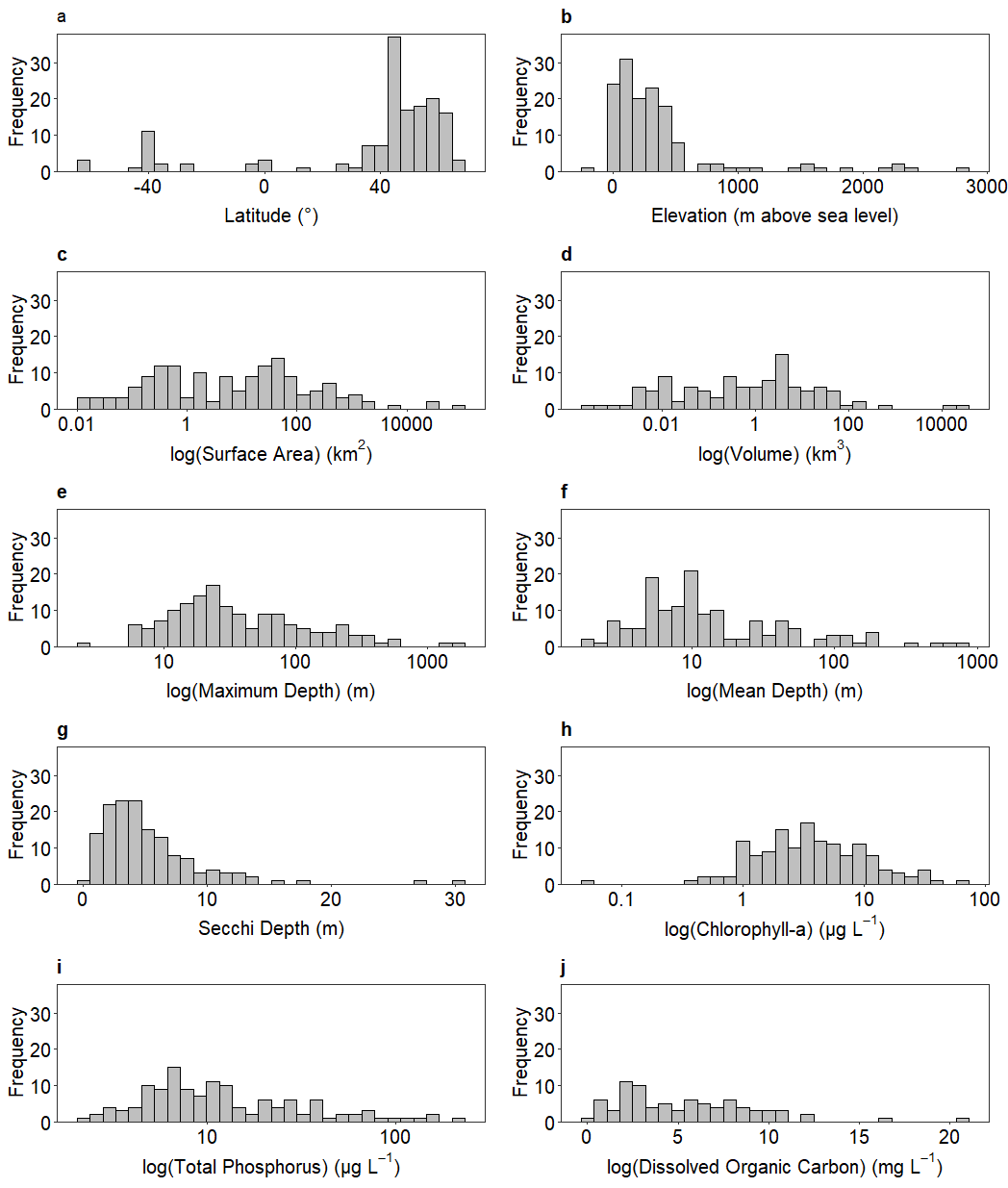
SUPPLEMENTARY INFORMATION

**Figure S1.** Histograms for lake characteristic variables, including (a) latitude, (b) elevation, (c) surface area (log-transformed), (d) lake volume (log-transformed), (e) maximum depth (log-transformed), (f) mean depth (log-transformed), (g) Secchi depth, (h) chlorophyll-*a* (log-transformed), (i) total phosphorus (log-transformed), and (j) dissolved organic carbon (log-transformed).
